# *In vitro* digestive properties and the bioactive effect of walnut green husk on human gut microbiota

**DOI:** 10.3389/fmicb.2024.1392774

**Published:** 2024-08-16

**Authors:** Xiaolan Zhao, Jiabao Ying, Zhuochen Wang, Yulu Wang, Zhen Li, Tianyi Gu, Shujun Liu, Yulong Li, Bing Liu, Fengjiao Xin, Boting Wen

**Affiliations:** ^1^Laboratory of Biomanufacturing and Food Engineering, Institute of Food Science and Technology, Chinese Academy of Agricultural Sciences (CAAS), Beijing, China; ^2^Key Laboratory of Food Nutrition and Safety, College of Food Science and Engineering, Tianjin University of Science and Technology, Tianjin, China; ^3^Institute of Agro-Products Processing, Anhui Academy of Agricultural Sciences, Hefei, China; ^4^Laboratory of Biomanufacturing and Food Engineering, Institute of Agricultural Product Processing and Nutritional Health, Chinese Academy of Agricultural Sciences (CAAS), Cangzhou, China

**Keywords:** walnut green husk, *in vitro* digestion, *in vitro* fermentation, gut microbiota, metabolism

## Abstract

**Introduction:**

Walnut green husk (WGH) is a waste byproduct from walnut industry. However, it is not well-known about its bioactive effect on human gut health.

**Methods:**

This study conducted *in vitro* digestion and fermentation experiments to study the bioactive effect of WGH.

**Results:**

Microbial fermentation was the primary mechanism to efficiently release phenolics and flavonoids, resulting in more excellent antioxidant capacities (DPPH, ABTS, and FRAP assays), which reached a highest value with 14.82 ± 0.01 mg VcE/g DW, 3.47 ± 0.01 mmol TE/g DW, and 0.96 ± 0.07 mmol FeSO_4_·7H_2_O/g DW, respectively. The surface microstructure of WGH became loose and fragmented after microbial fermentation. The analytical results of gut microbiota demonstrated that WGH could significantly increase the relative abundance of Proteobacteria in phylum level and *Phascolarctobacterium* in genus level while certain pro-inflammatory bacteria (such as *Clostridium_sensu_stricto_1, Dorea*, *Alistipes*, and *Bilophila*) was inhibited. Additionally, 1,373 differential metabolites were identified and enriched in 283 KEGG pathways. Of which some metabolites were significantly upregulated including ferulic acid, chlorogenic acid, umbelliferone, scopolin, muricholic acid, and so forth.

**Discussion:**

These results indicated that WGH could have antioxidant and anti-inflammatory activities in the human gut, which could improve the economical value of WGH in the food industry.

## 1 Introduction

Walnut (genus: *Juglans*), as the first of the four major dry fruits, is the second largest oil seed crop in China. Walnuts are mainly made up of kernels, green husk, shells, and diaphragma juglandis fructus. Walnut green husk (WGH), the unripe green pericarp of walnut fruit, as the by-product from walnut harvest processing, is rarely researched for its potential value. Current researches on WGH have focused on its composition and function. WGH represents a natural source of polyphenols, flavonoids, quinones, and other beneficial components (Soto-Madrid et al., 2021). It was reported to have potential antioxidant, antimicrobial, anticancer, and antiviral biological activities and the like (Alshatwi et al., 2012; Ahmadi et al., 2023; Xi et al., 2023). However, WGH has limited application areas, which is mainly related to dye and heavy metal removal and natural hair dyeing (Jahanban et al., 2019).

Human gut provides a good living environment for hundreds of microbial species, which play important physiological roles in immunity, nutrition, and metabolism (Passos and Moraes-Filho, 2017). A health body’s state is closely related to a healthy gut. Gut microbiota is sensitive to external environment and is easily susceptible to various factors, such as age, gender, lifestyle, and diet (Abdollahi-Roodsaz et al., 2016). Of these, diet is a crucial factor. Gut microbiota can ferment and utilize substances that remain undigested in the upper gastrointestinal tract to synthesize various bioactive metabolites including short-chain fatty acids (SCFAs), phenolics, and flavonoids (Leeuwendaal et al., 2022). These bioactive metabolites can further influence metabolism, host immunity responses, etc., through direct or indirect regulation mechanisms, such as polymethoxyflavones from citrus can help reduce the weight of body (Zeng et al., 2020); Isoquercitrin, existed in many herbs such as mulberry leaves and rooibos, can help to inhibit the transportation of tryptophan into bacteria to decrease the production of indole in the gut (Wang et al., 2020). However, it is unclear of the metabolic mechanism and the effect of WGH on human gut microbiota, which needs to be further studied.

At present, the studies focused on gut health are mainly conducted by *in vivo* and *in vitro* tests. *In vivo* studies (animal or human) can effectively elucidate the effect of the bioactive materials on mammals, while they are costly, inconvenient, and ethically restricted (Sensoy, 2021). In contrast, although *in vitro* studies such as non-stirred gut model may not mimic the complexity of the digestive system in the body due to the influence of various factors such as enzymes, absorption, secretion into the intestinal lumen, and interaction between the living intestinal wall and the microbiota*, in vitro* experiments can offer a low-cost, convenient, effective and ethically unrestricted alternative (Reboredo-Rodríguez et al., 2021). Xie et al. (2022) studied human gut microbiota in non-stirred gut model, and the results showed that human gut microbiota could utilize mung bean coat to release polyphenols, thus potentially contributing to gastrointestinal and colonic health. Ye et al. (2023) investigated *in vitro* that hypsizygus marmoreus polysaccharides could modulate the composition of gut microbiota and could be considered a potential prebiotic candidate. Ge et al. (2022) studied that insoluble dietary fiber from bamboo could be partially utilized by specific bacteria in human intestines in *in vitro* model and regulate the composition and microbial diversity of gut microbiota. Additionally, the complete digestive process of food is divided into two main parts: the upper gastrointestinal tract and microbial fermentation (Lucas-González et al., 2018; Ji et al., 2021), so a comprehensive study of *in vitro* upper gastrointestinal tract and microbial fermentation can effectively understand the correlations between food and gut microbiota.

This study employed *in vitro* digestion and fermentation experiments using WGH to achieve the following objectives: (1) determine the antioxidant capacity, micromorphology, and SCFAs levels; (2) study the effect of WGH on gut microbiota; and (3) identify and evaluate the metabolites from WGH. This study explored the digestive properties of WGH under simulated *in vitro* digestion conditions, such as its stability in gastrointestinal tract and the release of polyphenols. Meanwhile, it also explored the effect of WGH on gut microbiota, including promoting beneficial microbiota and inhibiting harmful microbiota. This work could provide suggestions for elucidating the metabolic mechanism of WGH and also provided theoretical support for the research and development and production of WGH-related nutritious health food, which would improve its additional value and expand the application field.

## 2 Materials and methods

### 2.1 Substrates and materials

The WGH was obtained from walnut tree cultivation (Beijing, China). WGH was washed and dried by oven (Shanghai, China) at 50°C for 48 h, then sieved through a 70mesh strainer after being crushed by a grinder.

The bile salt and digestive enzymes including α-amylase, pepsin, and trypsin were obtained from XiaoDong Pro-health Instrumentation Co. Ltd. (Suzhou, China). Simulated salivary, gastric and intestinal fluid (SSF, SGF and SIF) for *in vitro* digestion were made as reported by the literature (Pan et al., 2023). YCFA medium including yeast extract, tryptone, L-cysteine, NaCl, CaCl_2_·2H_2_O, K_2_HPO_4_, KH_2_PO_4_, MgSO_4_, heme, biotin, cobalamin, p-aminobenzoic acid, folic acid, pyridoxamine, thiamine, and riboflavin was obtained from Sigma-Aldrich (Sigma, United States). Gallic acid, Folin–Ciocalteu reagent, Na_2_CO_3_, and chromatography grade acetonitrile were purchased from Sigma-Aldrich (Sigma, United States). Rutin was obtained from Shanghai Aladdin Biotechnology Co. Ltd. (Shanghai, China). NaNO_2_, AlCl_3_·6H_2_O and sulfuric acid were obtained from Sinopharm Chemical Reagent Co. Ltd. (Beijing, China). PBS was obtained from Beijing LABLEAD Commercial and Trading Co. Ltd. (Beijing, China). SCFAs including formic acid, acetic acid, propionic acid, and butyric acid (chromatographic grade, purity ≥99.9%) were obtained from Shanghai Macklin Biotechnology Co. Ltd. (Shanghai, China). Other reagents and chemicals were analytically pure.

### 2.2 *In vitro* digestion

The procedure of *in vitro* digestion was performed as reported by Caicedo-Lopez et al. (2019) and Wang et al. (2019) with slightly modification. 50 g of WGH powder was dissolved in a 500 mL beaker containing 100 mL SSF and 100 mL distilled water, then at pH 7.0 and temperature 37°C for 10 min. Meanwhile, 250 mL SGF and 500 mL SIF were loaded into the simulated digestion model of the DHS-IV system provided by XiaoDong Pro-health Instrumentation Co. Ltd. (Suzhou, China) through injection tubes to simulate the retention of a certain amount of gastric juices in the stomach during the fasting state. The buccal digested chyme was loaded into a conical funnel and passed through the bionic esophagus within 5 min, and then the electromechanical apparatus was started. After gastric digestion and small intestine digestion for 2 h respectively, 10 mL of the digesta was collected from the bionic digestion model for subsequent phytochemical property analysis. All remaining small intestinal digesta was collected and lyophilized in a vacuum freeze-dryer provided by Beijing Songyuan Huaxing Technology Co. Ltd. (Beijing, China). After that, lyophilized powder was collected as the substrate for fermentation.

### 2.3 *In vitro* fermentation

*In vitro* fermentation was executed in accordance with the procedure described by Chen et al. (2020). The work employed was ethically reviewed and approved by the Institute of Food Science and Technology at the Chinese Academy of Agricultural Sciences (IFST-HREC-20240104). Human fresh feces were collected from 10 healthy volunteers (females: males = 1:1, aged 20–35 years old). Donors had not been exposed to antibiotics and pro/prebiotics or any other supplements for at least 2 months prior to sample collection. The informed consent was received from each donor. 5 g of fecal sample from each individual was dissolved in 50 mL of sterilized PBS solution. After full vortex shocking, the 10% fecal slurry suspension was filtered with 2 mL sterile syringes (Jiangsu Zhiyu Medical Equipment Co. Ltd.), respectively. The fermentation culture system included 15 mL YCFA (Schwab et al., 2017) and 0.15 g of digested lyophilized residues. The control was prepared with no digested lyophilized residues (CK). 1.5 mL filtrate was added and then anaerobic fermentation at 37°C started. 5 mL of fermentation broth was sampled at 0.3, 4, 8, 12, and 24 h. After centrifuging for 10 min at 9,500 × *g*, the precipitate was stored at −80°C for microbial analysis. The supernatant was employed for analyzing phytochemical bioactivity and SCFAs production. 10 mL of the remaining sample was stored at −80°C and then lyophilized for non-target metabolomic analysis.

### 2.4 Quantification of total phenolics and flavonoids content

The Folin–Ciocalteu test was employed to quantity the total phenolics content (TPC) (Dou et al., 2019), with slightly modification. 0.1 mL of sample supernatant was combined with 0.1 mL of Folin–Ciocalteu reagent, then, the addition of 0.15 mL of Na_2_CO_3_ water solution (w/v, 20%) and 0.75 mL of distilled water was performed. After thorough mixing, the mixed solution was left for 20 min at 37°C. The SpectraMax 190 light absorption enzyme labeler (Molecular Devices, United States) was used to determine the absorbance at 760 nm. The gallic acid was utilized to make a standard substance. Each sample was tested three times. The measurements were shown as milligram equivalents of gallic acid (GAE) per gram of dry weight (DW) materials.

The method reported by Qin et al. (2018) was utilized to quantity the total flavonoids content (TFC), with slightly modification. 100 μL of sample supernatant was added into 400 μL ethyl alcohol (v/v, 70%) and mixed thoroughly before adding 30 μL of NaNO_2_ solution (w/v, 5%). After 5 min of incubation at room temperature, 30 μL of AlCl_3_ solution (w/v, 10%) was blended and the mixed solution was maintained for 6 min. Subsequently, 200 μL of NaOH (1 M) and 240 μL of 70% ethyl alcohol were added. After that, the mixed solution was stand for 20 min at 37°C. The absorbance was determined at 510 nm. The rutin was utilized to make a standard substance. Each sample was tested three times. The measurements were shown as milligram equivalents of rutin (RE) per gram of dry weight (DW) materials.

### 2.5 Quantification of total antioxidant capacity

Total antioxidant capacity (T-AOC) was quantified by using 2,2′-azino-bis (3-ethylbenzthiazoline-6-sulfonic acid) (ABTS), 1,1-diphenyl-2-picrylhydrazyl radical (DPPH), and ferric reducing antioxidant power (FRAP) assays. The FRAP and ABTS analysis were conducted by using the T-AOC Assay Kit (S0116 and S0119, Beyotime, Shanghai, China). 5 μL of sample supernatant was blended with 180 μL of FRAP solution. After that, the absorbance value was measured at 593 nm. The FeSO_4_·7 H_2_O was utilized to make a standard substance. The FRAP assay measurements were shown as mmol FeSO_4_·7 H_2_O/g DW. 10 μL of sample supernatant was combined with 0.2 mL of ABTS radical cation solution and mixed thoroughly. After 6 min, the absorbance was measured at 734 nm. The Trolox was utilized to make a standard substance. The ABTS assay measurements were shown as mmol equivalents of Trolox (TE)/g DW. The DPPH Assay Kit (BC4755,100 T/48S, Solarbio, Beijing, China) was used to determine the DPPH value. 10 μL of sample supernatant was blended with 190 μL of DPPH working solution and mixed thoroughly. After standing at the dark environment for 30 min, the absorbance value was determined at 515 nm. The Vitamin C solution was utilized to make a standard substance. The DPPH assay measurements were shown as milligram equivalents of Vitamin C (VcE) /g DW. Each sample was tested three times.

### 2.6 Micromorphology analysis

The surface micromorphology of the sample after digestion and fermentation was discovered by employing a scanning electron microscope (SEM) (Hitachi, SU-8010, Japan). The operating voltage was 10.0 kV. About 3 mg of sample powder was pasted onto a sample stage using conducting adhesive and then sprayed with gold (90 s).

### 2.7 Analysis of pH and SCFAs

The concentration of SCFAs was analyzed by employing high performance liquid chromatography. 500 μL of sample supernatant was combined with 500 μL of chromatographic grade acetonitrile and mixed thoroughly. The mixed solution was centrifuged at 4°C and 9,500 × *g* for 30 min after maintaining for 10 min. After that, a 0.22 μm organic-based nylon filter membrane was used to filter the supernatant into the liquid phase vials and then the filtrate was tested online. Agilent 1220 system (Agilent Technologies) and an Aminex 300 mm × 7.8 mm column (HPX-87H, Bio-Rad Lt, Inc., United States) were employed. The flowing phase was made of sulfuric acid with a pH of 2.0. The flow velocity was scheduled to 0.6 mL/min. The wavelength was 210 nm. The temperature of the column oven was scheduled to 35°C. The volume of the loading sample was 10 μL. The formic, acetic, propionic, and butyric acid (chromatographic grade, purity ≥99.9%) were used to make the standard substances. A pH meter (Horiba, Japan) was employed to determine the pH.

### 2.8 Sequencing data analysis of gut microbiota

The precipitations of 10 experimental group samples and 10 control group samples after 24 h fermentation were delivered to Majorbio Bio-Pharm Technology Co., Ltd. (Shanghai, China) to conduct the sequence of bacterial 16 s rRNA, which was achieved on the Illumina Miseq sequencing platform (Illumina, San Diego, CA, United States). The V3-V4 variable area was targeted for sequencing by employing the Thermocycler PCR system (GeneAmp 9700, ABI, United States). According to a 97% similarity from SILVA database, the obtained sequencing data were grouped into operational taxonomic units (OTUs). Alpha diversity and community composition analysis were performed to comprehensively evaluate the microbial diversity (Quast et al., 2012). Bacterial universal primers are as follows:

806R (5′-GGACTACHVGGGTWTCTAAT-3′)
338F (5′-ACTCCTACGGGAGGCAGCAG-3′)

### 2.9 Non-targeted metabolomic analysis

20 fermented samples including 10 experimental group samples and 10 control group samples were lyophilized and delivered to Majorbio Bio-Pharm Technology Co., Ltd. (Shanghai, China) for untargeted metabolomics analysis using the LC–MS system. The Majorbio Cloud Platform was used to analyze data, including orthogonal partial least squares-discriminant analysis (OPLS-DA). The Variable Importance in Projection (VIP) analysis was conducted to determine the relative contribution of each variable, ranking their overall significance. According to the VI *p* value and the *p* value of the OPLS-DA model, differential expressed metabolites (DEMs) were screened. Significantly distinct metabolites were those having a VIP score of more than one and a *p* value of less than 0.05. The pathways connected with the different metabolites were found using Kyoto Encyclopedia of Genes and Genomes (KEGG) pathway database (Fang et al., 2022).

### 2.10 Data analysis

**Figure 1:**
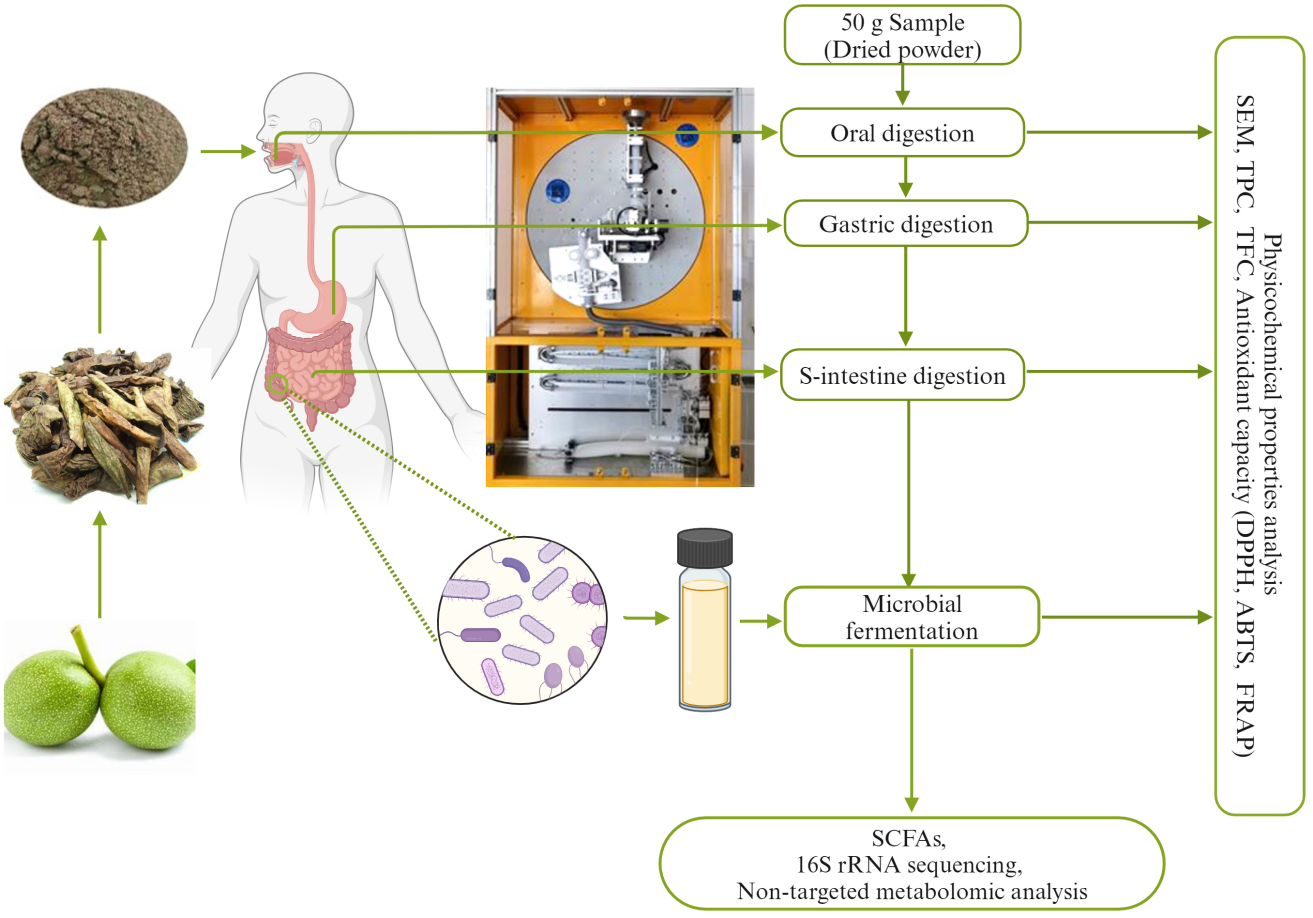
The whole digestion process map of walnut green husk (WGH).

The data on physicochemical properties were shown as the mean ± standard deviation (SD). The GraphPad Prism version 8.0 (GraphPad Software, La Jolla, CA, United States) was employed to visualize the data. SPSS 19.0 software was employed to conduct the significant analysis of data and Pearson correlation analysis between variables. The results were analyzed with the two-tailed unpaired Student’s *t*-test. 16S rRNA sequencing and non-targeted metabolomic analysis were performed on the Majorbio Cloud Platform. False discovery rate (FDR) correction was employed to address the issue of multiple comparisons and reduce the likelihood of false-positive results. The data was considered statistically significant when *p* value was less than 0.05 (Figure 1).

## 3 Results

### 3.1 Analysis of polyphenolic compounds levels during different digestion stages

**Figure 2:**
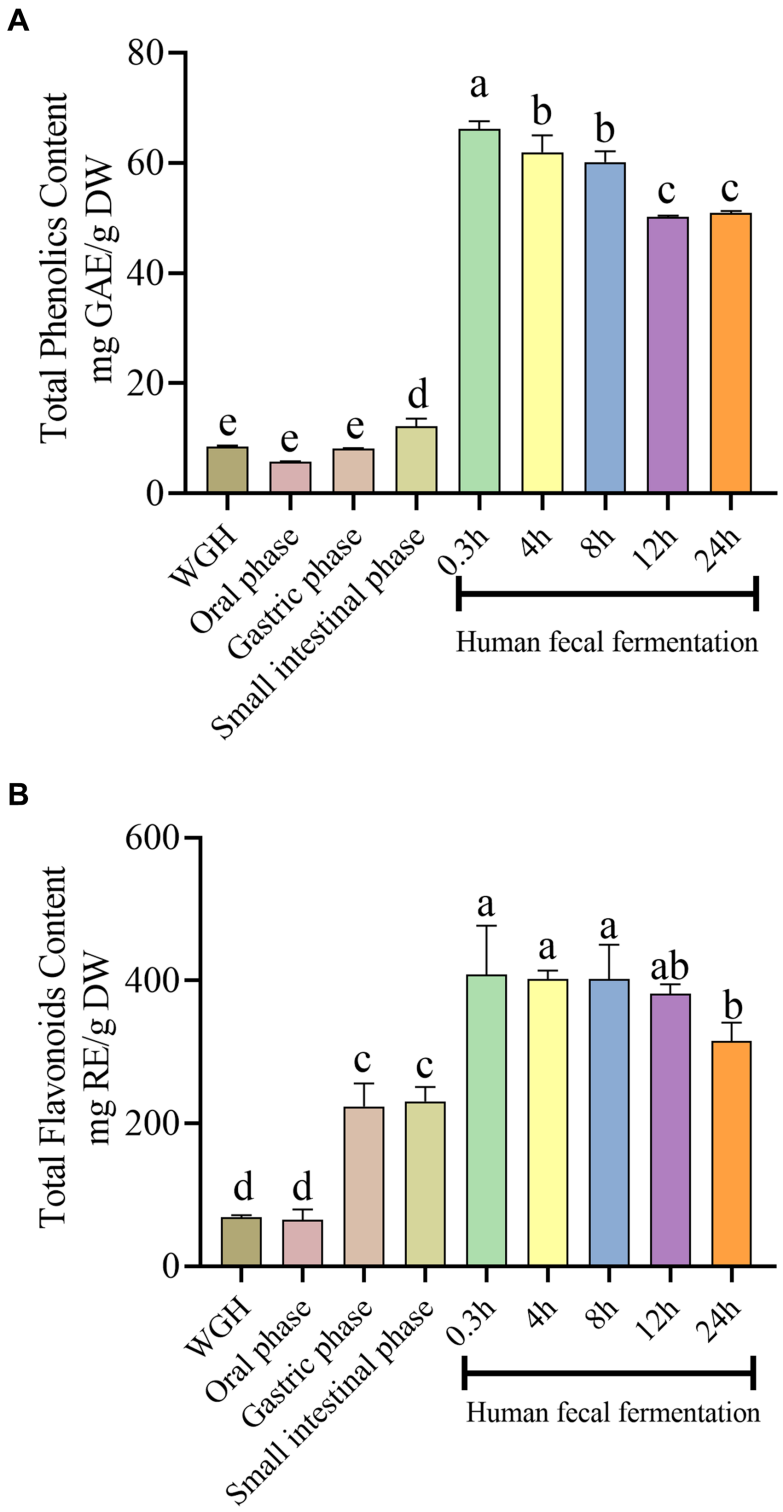
Effects of simulated digestion and fermentation on the released of phenolics and flavonoids from WGH in terms of **(A)** Total Phenolics Content (TPC); **(B)** Total Flavonoids Content (TFC). Un-digested samples were extracted with water. Bars with no letter in common are significantly different (*p* < 0.05).

To analyze the effects of oral-gastrointestinal digestion and fecal fermentation on the release of polyphenolic compounds, TPC and TFC were measured and results were shown in Figure 2. TPC of oral-gastroenteric digestion stage increased from 5.74 ± 0.04 mg GAE/g DW (oral phase) to 12.22 ± 0.09 mg GAE/g DW (intestinal phase). TFC of oral-gastroenteric digestion stage rose from 65.49 ± 0.25 mg RE/g DW (oral phase) to 230.96 ± 0.08 mg RE/g DW (intestinal phase). Surprisingly, TPC and TFC quickly reached the highest value with 66 ± 0.04 mg GAE/g DW and 408.38 ± 0.04 mg RE/g DW, respectively, at 0.3 h of fermentation, which was about five and two times of those in small intestine stage. During the fermentation stage, TPC gradually fell from 66 ± 0.04 mg GAE/g DW (0.3 h) to 50 ± 0.01 mg GAE/g DW (24 h), and TFC decreased from 408.38 ± 0.04 mg RE/g DW (0.3 h) to 315.22 ± 0.02 mg RE/g DW (24 h). Additionally, we found that TFC was overall higher than TPC, which was six times of TPC. Meanwhile, compared to undigested sample, TPC of the oral phase slightly decreased.

### 3.2 Analysis of antioxidant capacity during different digestion stages

**Figure 3:**
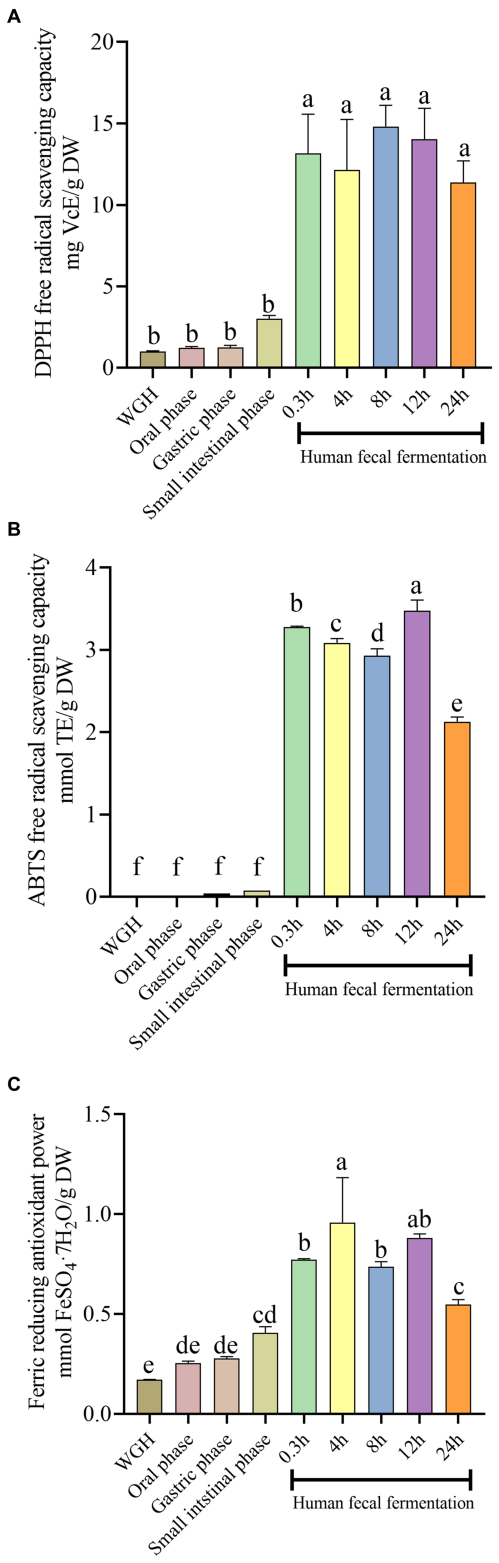
Effects of simulated digestion and fermentation on the antioxidant capacity of WGH in terms of **(A)** 1,1-diphenyl-2-picrylhydrazyl radical (DPPH) free radical scavenging capacity; **(B)** 2,2′-azino-bis (3-ethylbenzthiazoline-6-sulfonic acid) (ABTS) free radical scavenging capacity; **(C)** Ferric reducing antioxidant power. Bars with no letter in common are significantly different (*p* < 0.05).

In this study, DPPH, FRAP, and ABTS assays were employed to evaluate the antioxidant capacity at different digestive stages. As shown in Figure 3, the antioxidant capacity during the fermentation stage was greater than that during oral-gastrointestinal digestion and original stage, and showed the similar trend with TPC and TFC. Based on the Pearson correlation-coefficient analysis, we found that TPC and TFC were remarkably positively correlated with FRAP activity (*r* = 0.907, *p* = 0.001; *r* = 0.926, *p* < 0.001), ABTS^·+^ scavenging activity (*r* = 0.961, *p* < 0.001; *r* = 0.909, *p* = 0.001), and DPPḤ scavenging activity (*r* = 0.968, *p* < 0.001; *r* = 0.920, *p* < 0.001), suggesting that the metabolism of polyphenols in WGH might be positively correlated with its antioxidant capacity. However, the antioxidant capacity was decreased after 24 h fermentation, Whereas, the DPPH, ABTS, and FRAP values reached a highest value with 14.82 ± 0.01 mg VcE/g DW, 3.47 ± 0.01 mmol TE/g DW, and 0.96 ± 0.07 mmol FeSO_4_·7H_2_O/g DW, respectively.

### 3.3 Micromorphology analysis

**Figure 4:**
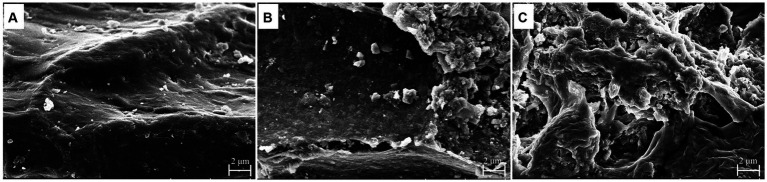
Changes in surface morphology of WGH before and after digestion and fermentation: **(A)** original; **(B)** after upper gastrointestinal digestion; **(C)** after fermentation.

Scanning electron microscope is a highly efficient method for observing and analyzing microstructural alterations. Therefore, it was employed to investigate the surface microstructure of WGH (Figure 4). As shown in Figure 4A, the original sample had a smooth, flat, hill-like surface. After upper gastrointestinal digestion, the sample showed a slightly curled lamellar structure, and the surface became rough with tiny holes, but most of the structure remained intact (Figure 4B). After being fermented by gut microbiota, the surface structure was severely destroyed and became fragmented (Figure 4C).

### 3.4 pH and SCFAs analysis

The pH and concentrations of SCFAs during fermentation were determined and summarized in Table 1. During 24 h fermentation, the pH decreased from 6.81 ± 0.09 (0 h) to 6.27 ± 0.25 (8 h), then increased to 6.54 ± 0.26 (24 h). The pH level was generally maintained between 6 and 7 and its fluctuation was not very dramatic. The concentration of total SCFAs exhibited an initial increase from 2.34 ± 0.65 g/L (0 h) to 4.94 ± 2.39 g/L (0.3 h), followed by a decrease to 1.86 ± 0.43 g/L (8 h), then increased to 3.73 ± 0.85 g/L (24 h), which might be broadly consistent with the trend in acetic and propionic acid levels. However, there was no butyric acid detected in the study, which differed from the findings of previous studies.

**Table 1:** The pH and SCFAs levels produced during *in vitro* fermentation of WGH.

Group	Formic acid (g/L)	Acetic acid (g/L)	Propionic acid (g/L)	Total SCFAs (g/L)	pH
0 h	0.22 ± 0.03^b^	1.56 ± 0.46^b^	0.56 ± 0.69^b^	2.34 ± 0.65^c^	6.81 ± 0.09^a^
0.3 h	0.12 ± 0.02^cd^	4.81 ± 2.40^a^	0.01 ± 0.00^c^	4.94 ± 2.39^a^	6.62 ± 0.15^ab^
4 h	0.07 ± 0.05^d^	4.24 ± 1.50^a^	0.17 ± 0.16^c^	4.49 ± 1.58^ab^	6.32 ± 0.32^c^
8 h	0.17 ± 0.07^bc^	1.06 ± 0.40^b^	0.63 ± 0.27^b^	1.86 ± 0.43^c^	6.27 ± 0.25^c^
12 h	0.22 ± 0.08^b^	1.12 ± 0.25^b^	0.82 ± 0.48^b^	2.16 ± 0.49^c^	6.42 ± 0.26^bc^
24 h	0.30 ± 0.12^a^	2.10 ± 0.55^b^	1.33 ± 0.49^a^	3.73 ± 0.85^b^	6.54 ± 0.26^b^

### 3.5 Analysis of the communities of gut microbiota

The alpha diversity of WGH and CK groups after fermentation was evaluated and shown in Supplementary Figure S1. The community abundance in samples is often estimated using chao and ace indexes. The community diversity is typically evaluated using the Simpson and Shannon indexes. In this study, WGH could significantly decrease the community diversity.

**Figure 5:**
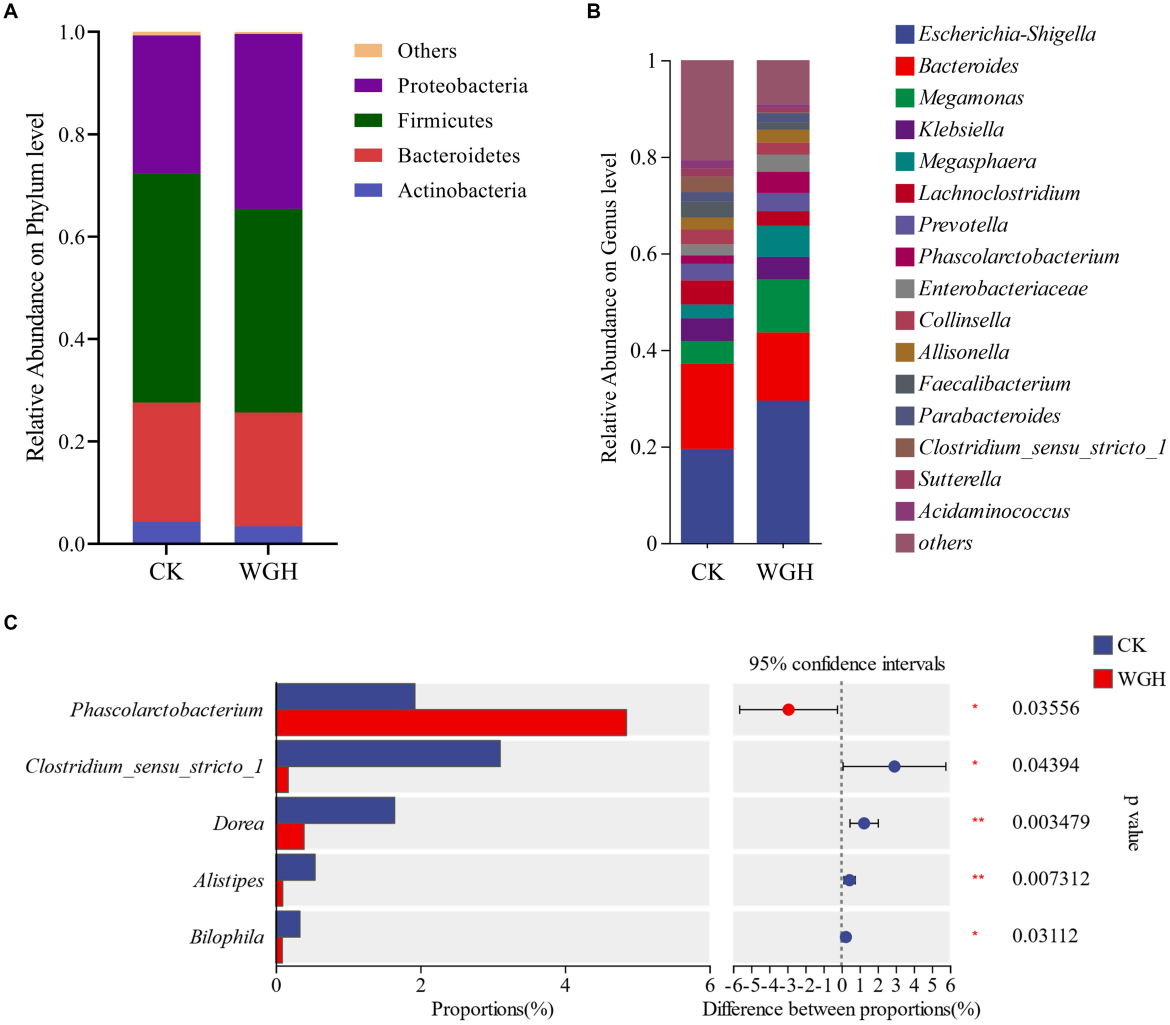
Effect of WGH on microbial species. **(A)** Microbial composition map in phylum level; **(B)** Microbial composition map in genus level; and **(C)** Analysis of significant differences in microbial composition in genus level (*p* < 0.05).

Figure 5 showed the relative abundance of microbial communities. In phylum level, Firmicutes, Bacteroidetes, Proteobacteria, and Actinobacteria were the dominant phyla (Figure 5A), accounting for 99% of intestinal microbiota. Additionally, WGH only increased Proteobacteria in phylum level. Figure 5B showed the relative abundance of microbial communities in genus level. The relative abundance of *Phascolarctobacterium*, *Megamonas*, *Prevotella*, and *Megasphaera* were increased in WGH group. Meanwhile, *Escherichia-Shigella* and *Enterobacteriaceae* were also observed an increasing trend in WGH group. Of these, only *Phascolarctobacterium* was significantly increased. However, *Clostridium_sensu_stricto_1*, *Dorea*, *Alistipes*, and *Bilophila* were significantly decreased (Figure 5C).

### 3.6 Analysis of the metabolite profiles after fermentation

**Figure 6:**
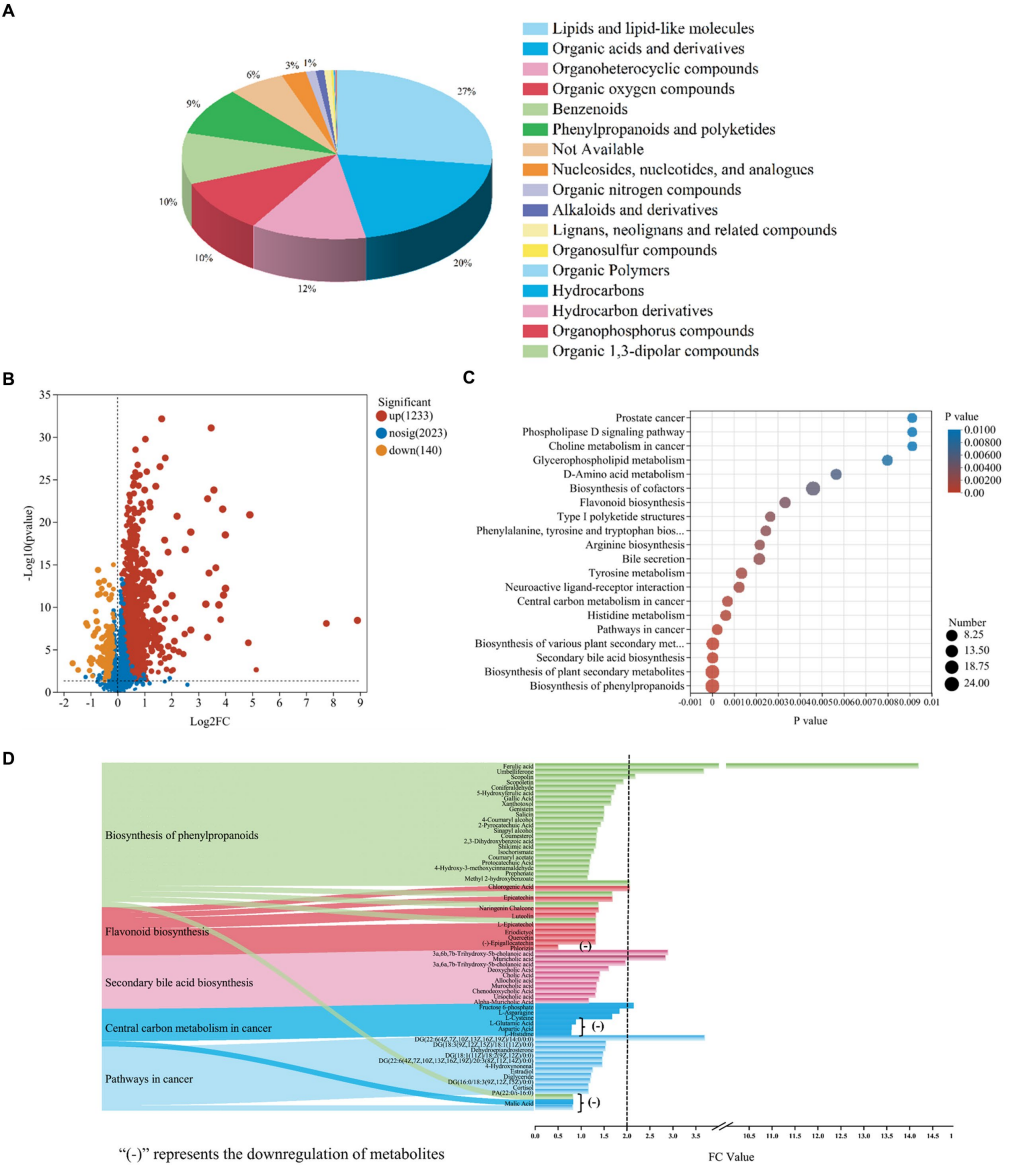
The metabolic profile of WGH after fermentation. **(A)** The pie chart of metabolites functional classification based on HMDB; **(B)** Volcanic map of differential metabolites between WGH and control groups; **(C)** KEGG enriched pathways of changed metabolites between WGH and control groups; and **(D)** Alluvial map and bar graph of significantly enriched pathways and metabolites correlations.

Walnut green husk could be further metabolized by gut microbiota to exert its physiological activities. The results were shown in Figure 6. Venn diagram showed that WGH and CK groups shared 2,144 metabolites besides there were 1,071 and 69 specific metabolites in the WGH and CK groups, respectively, (Supplementary Figure S2A). Furthermore, based on the analysis of OPLS-DA model, the metabolite composition between WGH and CK groups could clearly show distinct differences (Supplementary Figure S2B). According to the VIP analysis, DEMs were screened with the variables VIP > 1.0 and *p* < 0.05. There were 1,373 DEMs screened. These DEMs were classified as super-classes of lipids and lipid-like molecules (27%), organic acids and derivatives (20%), organoheterocyclic compounds (12%), organic oxygen compounds (10.3%), benzenoids (9.8%), phenylpropanoids and polyketides (9.1%), nucleosides, nucleotides, and analogs (2.5%) based on the Human Metabolome Database (HMDB) (Figure 6A). Volcano map showed that the WGH could lead to the upregulation of 1,233 metabolites and the downregulation of 140 metabolites (Figure 6B). The function of these changed metabolites was determined by KEGG pathway analysis. In the study, these metabolites were significantly enriched in 283 KEGG pathways. There were some important pathways including biosynthesis of phenylpropanoids, flavonoids, cofactors, plant secondary metabolites, and secondary bile acid, besides, pathways in cancer and central carbon metabolite in cancer (Figure 6C). The upward metabolites with fold change (FC) value above two were ferulic acid (14.19), DG [22:6(4Z,7Z,10Z,13Z,16Z,19Z)/14:0/0:0] (3.69), umbelliferone (3.67), 3a,6b,7b-trihydroxy-5b-cholanoic acid (2.89), muricholic acid (2.84), scopolin (2.18), fructose-6-phosphate (2.14), and chlorogenic acid (2.06) (Figure 6D). However, there were also some metabolites downward in certain pathways, such as phlorizin and L-glutamic acid as well as malic acid and aspartic acid.

## 4 Discussion

### 4.1 Analysis of total phenolic acids and total flavonoids content, antioxidant capacity, and surface micromorphology in different digestion stages

This study explored the effect of WGH on the human health. Phenolics and flavonoids are two main categories of polyphenolic compounds, which play important roles in the physiological regulations (de Araújo et al., 2021). In this study, parts of polyphenols could be released during the upper gastrointestinal digestion phase because of the combination blow of the acidic environment and digestive enzymes (Caicedo-Lopez et al., 2019). However, some phenolics might be degraded in the slightly alkaline, aerobic conditions (Tian et al., 2021). The involvement of microorganism fermentation and microorganism-secreted carbohydrate enzymes might contribute a lot on the bio-converting of free compounds and releasing of the conjugated phenolics and flavonoids from the complex polysaccharides (Zhang et al., 2019). During microbial fermentation, gut microbiota could also metabolize and utilize polyphenolic compounds (Yu et al., 2019). Free radicals are particles or clusters with unpaired electrons that are produced when covalent bonds of molecules are broken (Willcox et al., 2004). They tend to capture electrons from nearby molecules to make them stable, which can cause damage to the body (Zheng et al., 2022). Antioxidants can prevent the damage to cell tissue by binding free radicals. Polyphenolic compounds have excellent antioxidant capacity due to the capture of free radicals, which is beneficial to human health (Pérez-Burillo et al., 2018). This study used three assays to evaluate the antioxidant capacity of WGH, including ABTS, DPPH, and FRAP assays. Among three assays, antioxidants can provide ABTS^·+^ radicals with hydrogen atoms while DPPḤ and FRAP with single electron (Jin et al., 2012). Polyphenols might be metabolized slowly during fermentation (Zhang et al., 2023), resulting in a decrease in antioxidant capacity after 24 h fermentation. Moreover, polyphenolic compounds might undergo the structural modifications such as deprotonation and hydroxylation of hydroxyl groups (Bouayed et al., 2011). New compounds with higher capacities could be transformed as well (Cheng et al., 2019). The upper gastrointestinal digestion played minor roles in the metabolism and utilization of WGH, while the fermentation of gut microbiota was pivotal.

### 4.2 SCFAs analysis during fermentation

Human gut microbiota can fermentation carbohydrates and some bioactive compounds such as phenolics and flavonoids to produce SCFAs (Erickson et al., 2018), including formic, acetic, propionic, and butyric, which can not only provide energy for enterocytes but also contribute to maintaining gut homeostasis (Li et al., 2022). The production of SCFAs can result in the reduction of the pH of the gut, which helps to maintain a beneficial pH range of 6–7 in the human intestines (Zhou et al., 2022). In this study, WGH mainly promoted propionic acid and acetic acid in the human gut. However, butyric was not detected in this study, Ma et al. (2023) found that there was also no butyric acid produced after the fermentation of beechwood lignin-carbohydrate complexes and therefore speculated that this might be due to the structural variability of beechwood lignin-carbohydrate complexes with other plant-derived lignin-carbohydrate complexes. This result was consistent with the result of this study, so we speculated that this might be due to the poor utilization of the substrate by the gut microbiota due to the ubiquitous nature of the substrate.

### 4.3 Microbial diversity analysis

The results of 16S rRNA gene sequencing showed the effect of WGH on human gut microbiota. WGH could decrease the alpha-diversity of microbial community, which could be attributed to the competitive inhibition of dominant bacterial strains (Xu et al., 2023). WGH could promote the growth of some potentially beneficial bacteria and inhibit the proliferation of certain potentially detrimental microbiota. *Phascolarctobacterium*, *Megamonas*, *Prevotella*, and *Megasphaera* had been proven to be beneficial to the human health (Sheh, 2020). *Phascolarctobacterium*, one of the core genera of gut bacteria, could produce acetic and propionic acid by utilizing succinic acid (Wu et al., 2017), which was associated with the increasing of acetic and propionic level. Meanwhile, *Phascolarctobacterium* could alleviate intestinal inflammation and diarrhea, which was in accordance with the role of berberine (Chen et al., 2023). Huang et al. (2024) studied that *Megasphaera*, *Megamonas*, and *Phascolarctobacterium* might contribute to polyphenol liberation and metabolism in fermented quinoa, which was consistent with the results of this study. Meanwhile, the existence and digestion of polyphenols could promote the proliferation of *Prevotella* (Attri et al., 2018). However, the relative abundance of *Escherichia-Shigella* and *Enterobacteriaceae* were increased, which might be attributed to the mechanism of robust growth of these microflora (Wang et al., 2010; Baldelli et al., 2021). Certain bacteria such as *Clostridium_sensu_stricto_1*, *Dorea*, *Alistipes* and *Bilophila* were related to intestinal inflammation and some diseases (Huang et al., 2023), and their relative abundance were decreased in WGH. These results suggested that WGH could alleviate and reduce intestinal inflammation by modulating the composition of gut microbiota.

### 4.4 Non-targeted metabolomics analysis

The useful effects of WGH on the human body have been described. However, there have not been many reports on the effect of WGH on the composition and function of metabolites. According to the results of non-targeted metabolomics analysis, WGH could promote lots of functional metabolites that showed excellent antioxidant and anti-inflammatory potential. Ferulic acid was reported as an important antioxidant due to the capacity to scavenge free radicals and had been studied to have anti-inflammatory and antimicrobial activities (Chaudhary et al., 2019). Fang et al. (2024) studied that ferulic acid could improve intestinal barrier function by modulating the composition of gut microbiota. Kornicka et al. (2023) reviewed the pharmacological and therapeutic potential of umbelliferone including anti-inflammatory and anti-microbial effects, which could alleviate ulcerative colitis and microbial infections. It was reported that scopolin could alleviate hepatic steatosis induced by high-fat diet in mice (Yoo et al., 2017). Chlorogenic acid could alleviate irritable bowel syndrome by influencing the gut microbiota and its metabolites (Zheng et al., 2023). Additionally, as the intermediate product of the glycolytic pathway, fructose-6-phosphate could provide the energy required for the proliferation of cells (Brown et al., 2016). 3a,6b,7b-trihydroxy-5b-cholanoic acid and muricholic acid, belonging to secondary bile acid, might contribute to alleviating the metabolic syndrome and severity of colitis (Collins et al., 2022). DG [22:6(4Z,7Z,10Z,13Z,16Z,19Z)/14:0/0:0], as one of the members of diacylglycerol family, was proven to be involved in insulin resistance mechanisms (Lee et al., 2022). However, the mechanisms of action of some metabolites could be further studied.

Overall, our results suggested that the content of phenolics and flavonoids of WGH reached to maximum values with 66 ± 0.04 mg GAE/g DW and 408.38 ± 0.04 mg RE/g DW after fermentation. WGH significantly enriched *Phascolarctobacterium* and inhibited some pro-inflammatory bacteria. Based on the differential metabolome analysis, metabolites with a FC value of more than two included ferulic acid, umbelliferone, chlorogenic acid, and so forth, which were enriched in the function pathways including biosynthesis of phenylpropanoids, flavonoids and pathways in cancer. This study revealed that WGH seldom performed functions in gastrointestinal digestion while it could exert anti-inflammatory and antioxidant activities by modulating the composition of gut microbiota and enriching many functional metabolites. In future, functional food products or dietary supplements enriched with WGH can be developed to promote gut health, modulate microbial composition and potentially mitigate inflammation and oxidative stress-related conditions. Moreover, the findings of the study may contribute to the development of personalized nutrition strategies targeting gut microbiota modulation for improving health outcomes. However, the sample population for this study may be limited in terms of audience size, which may not reflect the gut microbiota of the entire population. Meanwhile, multi-omics analyses such as macro-genomic analysis, macro-transcriptomic analysis, and proteomic analysis can be employed to further explore the effect of WGH on gut microbiota.

## Data availability statement

The original contributions presented in the study are included in the article/Supplementary material, further inquiries can be directed to the corresponding authors.

## Ethics statement

The studies involving humans were approved by Ethical Review Committee of the Institute of Agricultural Products Processing, Chinese Academy of Agricultural Sciences. The studies were conducted in accordance with the local legislation and institutional requirements. The participants provided their written informed consent to participate in this study.

## Author contributions

XZ: Data curation, Investigation, Resources, Validation, Visualization, Writing – original draft. JY: Resources, Writing – review & editing. ZW: Writing – review & editing. YW: Writing – review & editing. ZL: Funding acquisition, Writing – review & editing. TG: Methodology, Writing – review & editing. SL: Formal analysis, Writing – review & editing. YL: Methodology, Writing – review & editing. BL: Supervision, Writing – review & editing. FX: Supervision, Writing – review & editing. BW: Conceptualization, Funding acquisition, Project administration, Supervision, Writing – review & editing.
