# Supplementary material for: In vitro digestive properties and the bioactive effect of walnut green husk on human gut microbiota
Source: Front Microbiol. 2024 Aug 16;15:1392774. doi: 10.3389/fmicb.2024.1392774 (PMC11367867; doi:10.3389/fmicb.2024.1392774)
Supplement: Supplementary file 1 [file Table_2.DOCX]

Supplementary Material


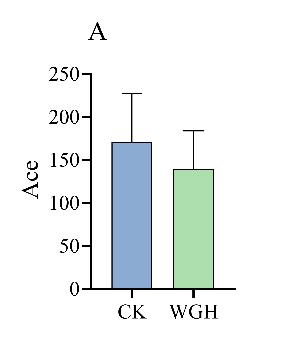

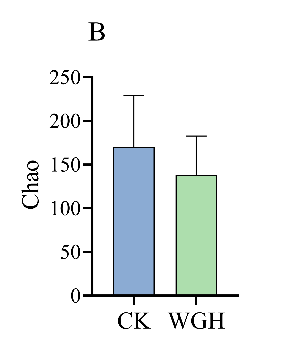

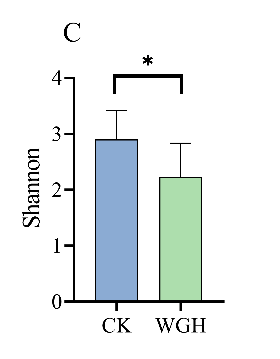

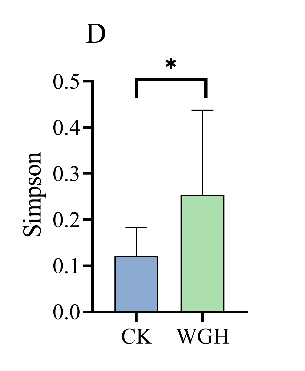


**Supplementary Figure 1.** Effect of walnut green husk on microbial alpha-diversity. (A) Ace index;(B) Chao index; (C) Shannon index; (D) Simpson index.


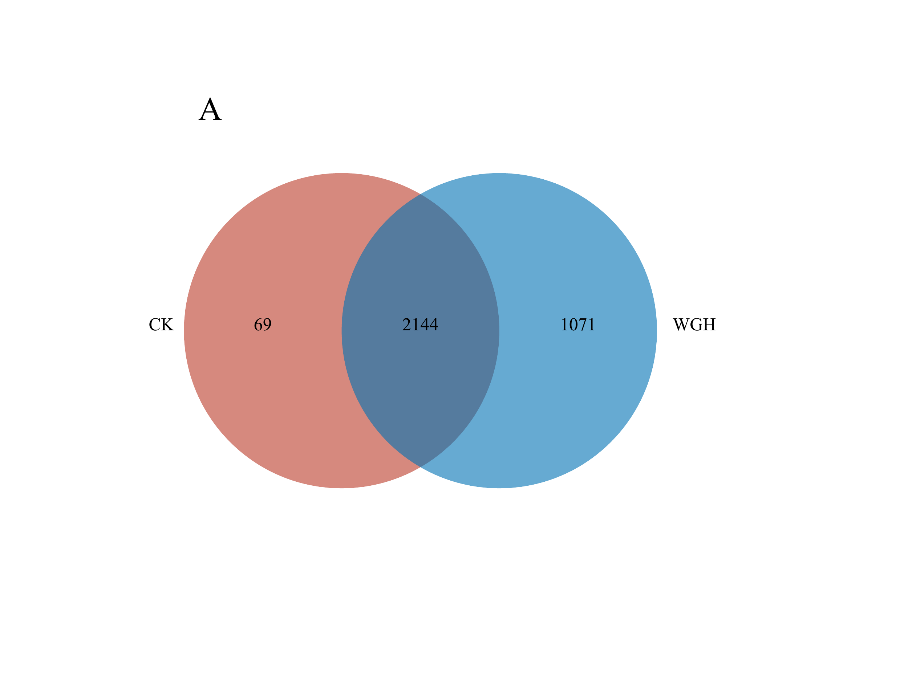

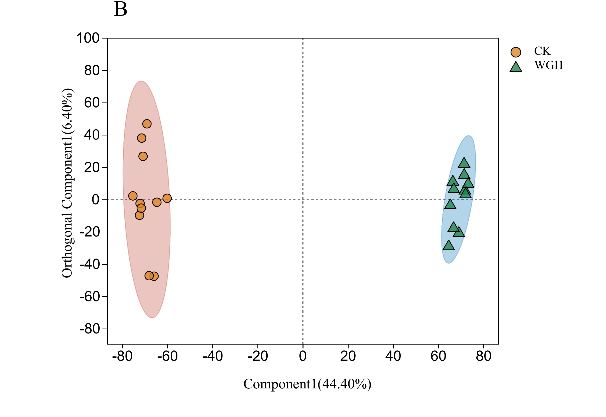


**Supplementary Figure 2** The metabolic profile of walnut green husk after fermentation. (A) Venn diagram of metabolites between WGH and CK groups; (B) OPLS-DA score map in the mix ion mode.
